# Not All Continuous Dimensions Map Equally: Number-Brightness Mapping in Human Infants

**DOI:** 10.1371/journal.pone.0081241

**Published:** 2013-11-20

**Authors:** Maria Dolores de Hevia, Elizabeth S. Spelke

**Affiliations:** 1 Université Paris-Descartes, Laboratoire Psychologie de la Perception, CNRS, UMR 8158, Paris, France; 2 Cognitive NeuroImaging Unit, NeuroSpin, INSERM, U992, Gif sur Yvette, France; 3 Laboratory for Developmental Studies, Harvard University, Cambridge, Massachusetts, United States of America; University of Bath, United Kingdom

## Abstract

Evidence for spontaneous mappings between the dimensions of number and length, time and length, and number and time, has been recently described in preverbal infants. It is unclear, however, whether these abilities reflect the existence of privileged mappings between certain quantitative dimensions, like number, space and time, or instead the existence of a magnitude system underlying the representation of any quantitative dimension, and allowing mappings across those dimensions. Four experiments, using the same methods from previous research that revealed a number-length mapping in eight-month-old infants, investigated whether infants of the same age establish mappings between number and a different, non-spatial continuous dimension: level of brightness. We show that infants are able to learn and productively use mappings between brightness and number when they are positively related, i.e., larger numbers paired with brighter or higher contrast levels, and fail when they are inversely related, i.e., smaller numbers paired with brighter or higher contrast levels, suggesting that they are able to learn this mapping in a specific direction. However, infants not only do not show any baseline preference for any direction of the number-brightness mapping, but fail at transferring the discrimination from one dimension (number) to the other (brightness). Although infants can map multiple dimensions to one another, the number-length mapping may be privileged early in development, as it is for adults.

## Introduction

Representations of quantitative dimensions share fundamental cognitive and neural relations. Psychophysics research established long ago the effortless ability of human adults to translate any quantitative dimension into any other, for instance mapping loudness level onto handgrip pressure [1], [2]. This intuitive mapping of the type ‘more a, more b’ has been also noted in developmental contexts, with children estimating, for instance, that a longer train moves faster than a shorter one, even if they both run at the same speed [3], or that a brighter light lasts longer than a dimmer one that is presented for the same duration [4]. Classical neuropsychological studies have provided more evidence for common processing of diverse magnitudes, including numbers. In fact, since the description of Gerstmann syndrome [5] the co-occurrence of deficits in arithmetic, spatial and abstract perceptual judgments has been highlighted. More recent research has shown that overlapping brain areas in the parietal cortex are involved in the processing of different quantitative dimensions, such as number and spatial extent [6], number and spatial orientation [7], [8], and possibly other non-spatial dimensions such as brightness [9].

One of the main cognitive attributes characterizing representations of quantitative dimensions is their analog format [10]. This signature of magnitude dimensions reflects the fact that discrimination for any of these continua conforms to Weber’s law, indicating that a successful discrimination between two quantities depends on their ratio rather than on their absolute values [10], [11], [12]. Many perceptual dimensions have been found to follow this representational constraint, including number [12], spatial extent [13], brightness [1], loudness [1], and even more abstract dimensions such as the ferocity or intelligence of animals [14]. Thus, the cognitive and/or neural constraints appear to be similar in the representations of any attribute that can be formalized in ‘more than’ or ‘less than’ terms.

The link between numbers and space has been one of the most prominent and studied relationships between quantitative dimensions. A vast literature illustrates the phenomenon by which visuospatial resources are recruited whenever processing of number occurs, shedding light on the representational format of numerical representation. For example, the Stroop paradigm, conducted with the dimensions of size (or spatial extent) and number, reveals that variation on one dimension, irrelevant to the task at hand, influences the judgment of the other dimension: deciding which of two numbers is numerically bigger is either facilitated or impaired, depending on whether the physical size with which the numbers are presented is congruent or incongruent with their numerical size [15], [16]. This phenomenon is bidirectional, so that judgments of physical size are affected by numerical size, and judgments of numerical size are affected by physical size. As a second example, the observation of the SNARC (Spatial Numerical Association of Response Codes) effect points to the automatic activation of an oriented spatial continuum when processing numbers: small numbers are associated to the left side of space and large numbers to the right side of space, in particular in Western cultures with a left-to-right oriented reading/writing system [17], [18]. Finally, vision does not appear to be a prerequisite for the spatial format of number to emerge, since congenitally and early blind subjects show the same SNARC phenomenon as sighted participants do [19].

The psychological links between space and time also are well known. We co-opt spatial language to refer to temporal terms, and temporal judgments are affected by the spatial dimension [20]. Research investigating commonalities between representations of different quantitative dimensions has been inspired by the seminal studies on the common representation of time and number in rats [21]. For instance, when adults judge the duration of stimuli that vary in size, brightness and number, they make longer temporal estimations with increasing magnitudes across these dimensions [22]. These findings raise the possibility that a single system of magnitude represents these, and possibly any, dimensions of quantity [10], [23], [24]. In other words, magnitude representations may be rooted in a single developmental algorithm for ‘more than/less than’ distinctions of any variable in the external world [23]. In support of this view, adults are able to map number onto spatial [25] as well as a variety of non-spatial formats, such as squeezing, bell striking and vocalizing [26].

A critical source of evidence bearing on this view comes from studies of infants and young children who lack formal education and have minimal experience with language and other symbol systems. Research on infants has shed new light on the developmental origins of quantitative representations and of their relations. Some evidence suggests a unified magnitude system for the dimensions of number, space and time. First, the developmental literature has established parallelisms in the precision with which infants represent magnitude changes in the domains of number, area or spatial extent, and time. Six-month-old infants require a 2:1 ratio in order to discriminate instances across the domains of number [27], [28], time [29], [30] and size [31]; 9-month-old infants require a 3:2 ratio for all these dimensions [27], [29]. Second, we now know that 8- and 9-month-old infants are able to link the representations of number, spatial extent and temporal duration, with infants creating number-length mappings [32], [33], number-time mappings [33], and time-length mappings [34], and even 3 to 4-week-old infants are able to create cross-modal mappings across the dimensions of brightness and loudness [35].

Some studies suggest, however, that links between the dimensions of number, space and time might have a more prominent status than mappings between other dimensions, for both adults and children, and even for preverbal infants. On the one hand, while adults show robust bidirectional interference in a Stroop task for the dimensions of number and size, and brightness and size, the interference between number and brightness is unidirectional, with brightness mildly interfering with number but not the reverse [6]. These differences in interference patterns suggest that the three dimensions of number, size and brightness are not processed identically. On the other hand, preschool children reliably form mappings between the dimensions of number and spatial extent, are partially accurate in establishing mappings between spatial extent and brightness, but fail completely at creating mappings between number and brightness [36]. Moreover, preverbal infants, at 9 months of age, fail to create mappings between spatial length and loudness, whereas they succeed with the mapping between spatial length and temporal duration [34].

The literature therefore points to the existence of shared mechanisms for processing numerical, spatial and temporal magnitudes from early in infancy, but is ambiguous regarding adults’ abilities to create mappings among any dimensions. The present research investigates whether infants' mappings of number to other quantitative dimensions extend to other, less canonical quantitative dimensions: levels of brightness and contrast. We adopted the same four methods and materials as in de Hevia & Spelke's (2010) studies of mappings of number to length. Instead of presenting lines of different lengths onto which numbers could be mapped, we presented forms that differed in both brightness and contrast. In Experiments 1 and 2, we tested whether infants are able to learn and productively use a rule relating number to brightness/contrast. In Experiment 1 we tested infants with a positive pairing of number and brightness (where larger numbers are accompanied by brighter, higher contrast objects); in Experiment 2 we tested infants with an inverse pairing of number and brightness (where larger numbers are accompanied by darker objects with lower contrast). Experiment 3 tested whether infants show a baseline preference for either of the two types of number-brightness mappings. Previous research has shown both that infants prefer positive number-length pairings (when tested by the method of Experiment 3) and that they generalize positive but not inverse pairings to new exemplars (when tested with the methods of Experiments 1 and 2). In contrast, the present research provided no evidence that infants prefer positive number-brightness pairings, and only weak and partial evidence that infants preferentially learn positive number-brightness pairings. Finally, in Experiment 4 we tested whether infants transfer the discrimination of an ordered series of numbers to the discrimination of an ordered series of brightness (or contrast) levels. At the same age, infants are able to do this task for ordered series of numbers and lengths [32], but they fail to do so for the present displays mapping number to brightness.

## Experiment 1

**Figure 1:**
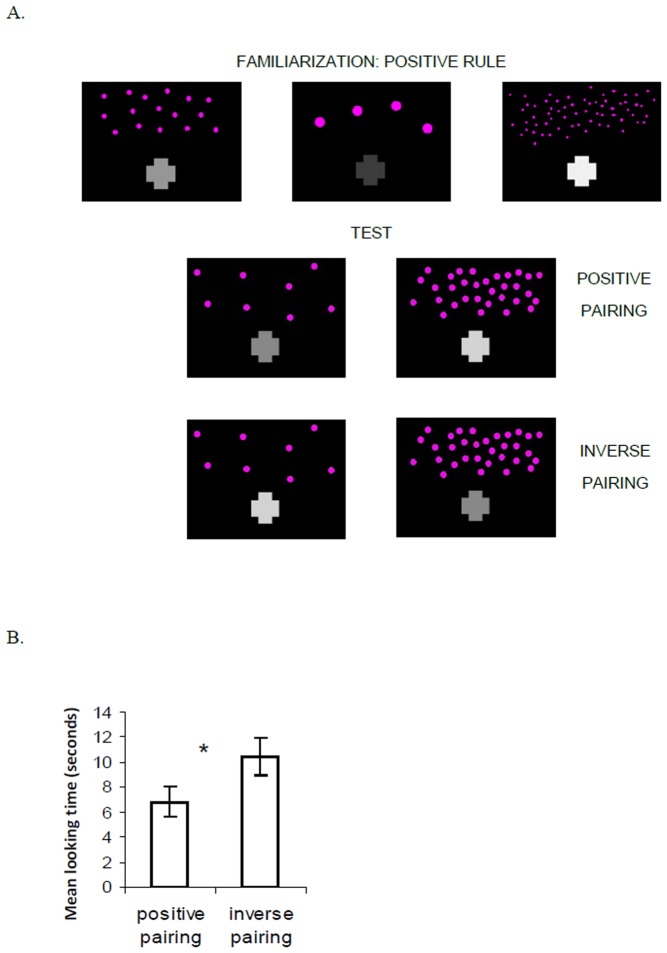
Displays used in the familiarization and test phases for Experiment 1, and mean looking times during test trials. A. Examples of displays used in the familiarization phase with a positive number-brightness pairing, where larger numbers are accompanied by brighter objects. In test, infants are shown new numbers and new brightness levels, either in a positive pairing where larger numbers are accompanied by brighter and higher contrast objects, or in an inverse pairing where larger numbers are accompanied by darker or lower contrast objects. B. Mean looking times (seconds) to the positive and the inverse test pairing trials. The asterisk denotes a significant difference between looking times to each pairing.

In this experiment, infants were presented with a succession of displays containing a set of visual elements (e.g. dots) above a cross. Although both number and brightness changed randomly from one display to another, the two dimensions were positively correlated: larger numbers were accompanied by brighter forms with higher contrast levels. To test whether infants would learn this correlation and generalize it to new numerical and brightness values, infants first were habituated to these arrays and then were shown two test trials presenting sequences with new numbers and brightness/contrast levels that were paired either following the familiar rule (i.e., higher brightness/contrast levels accompanying greater numbers) or a novel rule (i.e., lower brightness/contrast levels accompanying greater numbers; Fig. 1A). If infants extracted the rule that higher number was related to higher levels of brightness/contrast, they should have applied that rule to the test exemplars and should discriminate between new pairings conforming to the extracted rule over new pairings that did not conform to the rule. In research using this method with positive number-length pairings, infants of this age expressed their rule learning by looking longer at the new test displays that conformed to the rule [32].

### Materials and Methods

#### Participants

Participants were 20 infants (10 female and 10 male; mean age  =  8 months, range: 7 months 15 days to 8 months 15 days). Three other infants were eliminated because of crying (2), or parental interference (1).

#### Ethics Statement

The experiment was conducted after obtaining Institutional Review Board approval from the Department of Psychology at Harvard University. All participants' parents gave informed written consent before testing began.

#### Materials

The familiarization displays were made by combining a cross presented at three levels of brightness, and contrast with the background (i.e., a cross whose gray level included 20%, 60% or 100% white, presented against a black background), and an array of elements presented at three numerical magnitudes (i.e., 4, 16, and 64 colored forms, presented against the black background). The cross subtended an area of 4.4° visual angle horizontally and 3.9° vertically. The brightest display both had the highest luminance and the greatest brightness contrast, since the latter has been found to determine the psychological direction of the continuum: larger the contrast are associated with larger numbers [37] and longer lines [36]. Numerical displays were composed of colored circles, squares, or equilateral triangles. For each trial, only one type of figure and one color were presented. The total overall area of the visual elements was kept constant across numerosities: For arrays of 4, 16, and 64, respectively, circle diameters were 1.9°, 0.95°, and 0.45° visual angle; square sides were 1.7°, 0.85°, and 0.6° visual angle; and triangle sides were 2.4°, 1.2°, and 0.6° visual angle. Therefore, item size inversely correlated with number. As we employed the same numerical displays as in de Hevia & Spelke (2010), we changed the spatial properties of the numerical displays between familiarization and test. In the test displays, dot size was the same for all array sizes; number therefore was correlated only with summed area, a dimension that did not covary with brightness/contrast during familiarization. During familiarization, total overall area was constant across number arrays, and therefore the total brightness/contrast level was constant across numbers; during test, element size was kept constant. Therefore, the brightness/contrast level of individual elements was constant across numbers, and infants could not learn relations between brightness levels in the numerical array and in the cross appearing below that array. To discover the relationship between the two halves of each display, infants therefore needed to map abstract number to level of brightness.

The test displays consisted of an array of 8 dots and an array of 32 dots, each paired with a cross at a brightness level of 40% or at 80%. The dots were altered to keep their size constant (i.e., the dots’ diameters for numerosities 8 and 32 was 1.3° visual angle). Therefore, the numerical and brightness/contrast values presented during test were novel but lay within the range of the values presented during familiarization.

For each familiarization trial, three different images for each number-brightness pairing were presented in a pseudorandom order, so that consecutive numerosities did not follow any predictable order (e.g., 16, 64, 4, 16, 4, 64, 16, 4, 64). For each test trial, three different images for each of the two number-brightness pairings were presented in alternation, starting with 8 (i.e., 8, 32, 8, 32, 8, 32). The positions of the elements in the numerosity displays, which occupied the upper half of the screen, were varied across trials, while the cross was always horizontally centered in the lower half of the screen. Each familiarization and test trial consisted of a repeating cycle: A numerical array, centered in the upper half of the screen (1000 ms), was subsequently joined by a cross centered on the lower half of the screen (1000 ms); this stimulus was followed by a blank screen (500 ms) and then the next display. Each cycle lasted 22.5 s during familiarization and 15 s during the test; cycles were looped until the end of each trial.

#### Design

Infants were familiarized with the number-brightness pairings (i.e., larger numbers accompanied by a brighter cross), and then were tested with new pairings following either the familiar or the new, inverse rule (i.e., larger numbers accompanied by a darker cross). Half the infants were tested on the familiar pairing rule first, and the other infants were tested on the novel, inverse pairing rule first.

#### Procedure

Infants were seated on a parent’s lap in a softly illuminated room and faced a screen surrounded by black surfaces and curtains. Parents were instructed to refrain from interacting with their infants and to close their eyes during the test sequences. A video camera below the screen was directed at the infant’s face, and a second video camera (display camera) was placed behind the infant to record the displays. The footage from the two video cameras was sent to a TV monitor and a VCR in a separate room, where one or two observers recorded the infant’s looking times. During this coding process, the display footage was occluded to ensure that the observer was blind to the habituation and test conditions. For 14 of the 20 infants, two observers coded the data live or from videotape; average intercoder reliability was 91%.

At the beginning of each trial, a black occluder was lifted to reveal a black screen (65 cm×40 cm) on which images were presented; the total display area measured 28.4°×18.4° of visual angle at a viewing distance of about 60 cm. Each display remained visible until the infant looked for at least 0.5 s, and ended when the infant looked away for 2 s continuously (or looked for a maximum of 120 s). Familiarization trials continued until the infant either received 14 trials or reached the criterion of a 50% decline in looking time across 3 consecutive trials relative to the total looking time on the first 3 consecutive trials that had summed to at least 12 s. If an infant did not meet this criterion after the minimum possible 6 trials, the displays were cycled in the same order until habituation was achieved or the 14-trial limit was reached. Following familiarization, all infants were shown the two test displays.

#### Analyses

Test-trial looking times were submitted to an ANOVA with trial order (familiar rule vs. novel rule first) as the between-subjects variable and trial type (familiar vs. novel pairing rule) as the within-subjects variable. All other tests were two-tailed.

### Results and discussion

Infants received an average of 8.4 familiarization trials, exhibiting habituation from the first 3 trials (18.3 s) to the last 3 trials (8.7 s), *t*(19)  = 4.17, *p*<.001, paired-samples t-test. Relative to their performance in the last 3 familiarization trials, infants showed no dishabituation to the familiar, *t*(19) <1, n.s., or novel, *t*(19)  = −1.75, n.s., test displays, paired-samples t-tests. Still, infants looked longer at the novel test display (10.4 s) than at the familiar test display (6.8 s), *t*(19)  = −2.27, *p*  = .03, paired-samples t-test (Fig. 1B), and this effect was the only tested variable that affected looking times, *F*(1, 18)  = 4.96, *p* = .03. Thirteen out of 20 infants looked longer at the novel pairing during test (*Z* = 1.94, *p* = .052, Wilcoxon sign-ranked test; 2 infants looked equally to both test trials), while five infants looked longer to the familiar test pairing.

These findings provide some evidence that infants learned the number-brightness relationship in the familiarization displays and generalized this relationship to the new numbers and brightness levels in the test displays. During familiarization, the overall brightness level of the numerical arrays was constant whereas item size covaried with the brightness level of the cross; during test, the overall brightness level of the numerical arrays covaried with the brightness level of the cross but item size did not. Therefore, the rule that infants could have applied during familiarization was not available during test and vice versa, revealing that infants’ generalization depended on abstraction of a relationship between brightness/contrast level and element number. Thus, infants were sensitive to the number-brightness mapping in which larger numbers were accompanied by higher levels of brightness and contrast.

In the same testing conditions, previous research showed significantly higher looking times towards the familiar number-length test pairing in conditions where larger numbers were accompanied by longer lines during familiarization [32], whereas in the present study infants showed significantly higher looking times towards the novel number-brightness test pairing. It is possible that infants have a baseline preference for a pairing between number and brightness where larger numbers are accompanied by darker objects. Before examining baseline preferences in absence of a learning phase, Experiment 2 tested whether infants would also be able to learn and productively use a pairing between number and brightness where larger numbers are accompanied by darker objects, or lower levels of brightness.

## Experiment 2

**Figure 2:**
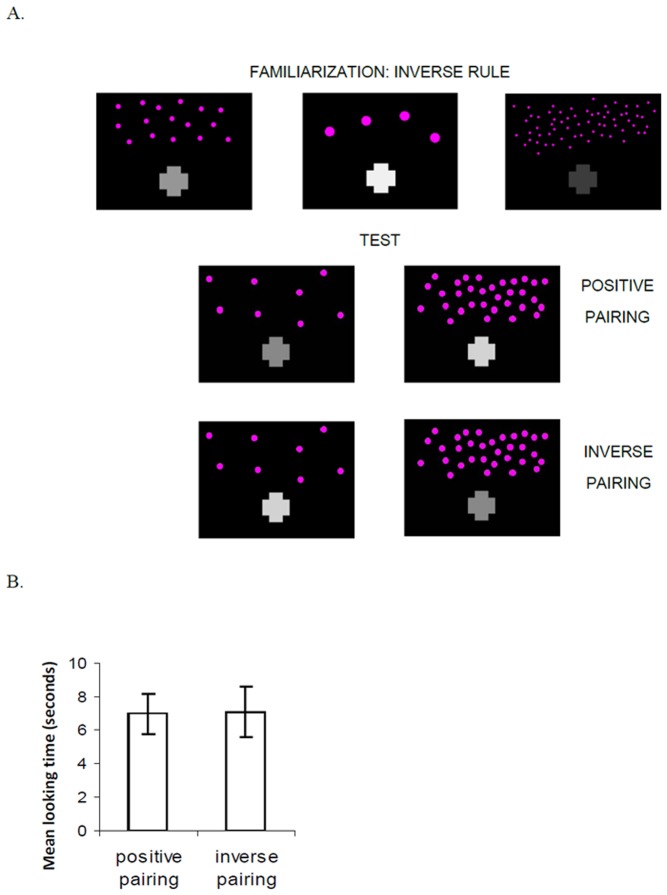
Displays used in the familiarization and test phases for Experiment 2, and mean looking times during test trials. A. Examples of displays used in the familiarization phase with an inverse number-brightness pairing, where larger numbers are accompanied by darker objects. In test, infants are shown new numbers and new brightness levels, either in a positive pairing where larger numbers are accompanied by brighter and higher contrast objects, or in an inverse pairing where larger numbers are accompanied by darker or lower contrast objects. B. Mean looking times (seconds) to the positive and the inverse test pairing trials. No significant difference was observed in the looking times to each pairing.

In experiments testing infants' sensitivity to number-length mappings, 8-month-old infants show no evidence of learning inverse number-length pairings, in which greater numbers are accompanied by shorter lines. When they were familiarized with these inverse pairings and tested with new positive and inverse pairings, they looked equally at the latter test displays. Moreover, infants’ performance at test reliably differed between the conditions showing the inverse pairing rule and the positive pairing rule, suggesting that the dimensions of number and length are mapped in a specific direction [32]. However, since from birth infants are able to learn arbitrary relationships between events [38], it is possible that infants will detect a relationship between number and brightness/contrast in either direction. In Experiment 2, we asked whether infants at the same age would show evidence of learning a number-brightness pairing rule during familiarization with higher brightness/contrast levels accompanying smaller numbers of visual elements (Fig. 2A).

### Method

The method was the same as in Experiment 1, except as follows. Participants were a new group of 20 infants (11 female and 9 male; mean age  =  7 months 29 days, range: 7 months 15 days to 8 months 13 days). Six additional infants were eliminated because of crying (3), technical error (1), or excessive test-trial looking times (2; more than 2.5 SD from the group mean). The familiarization arrays from Experiment 1 were presented in a consistent inverse relationship, such that the cross with the lowest brightness/contrast level accompanied the largest numerosity. For 16 of the 20 infants, two observers coded the data live or from videotape; average intercoder reliability was 95%.

### Results and discussion

Infants received an average of 8.5 familiarization trials and exhibited habituation from the first 3 trials (17.8 s) to the last 3 trials (6.6 s), *t*(19)  = 6.8, *p*<.0001, paired-samples t-test. Again, no dishabituation effects were observed for the new displays showing the familiar pairing rule, *t*(19) <1, n.s., or for the test displays showing the novel pairing rule, *t*(19) <1, n.s., relative to the familiarization displays (paired-samples t-tests). Infants also showed no preference between the test displays with familiar pairings (7.1s) and the test displays with novel pairings (6.9 s), *t*(19) <1, n.s., paired-samples t-test (Fig. 2B). Eight out of 20 infants looked longer to the novel test pairing, and 10 infants looked longer to the familiar test displays (*Z*<1, n.s., Wilcoxon sign-ranked test; 2 infants looked equally to both test pairings). The ANOVA revealed that the interaction between trial order (familiar vs. novel pairing first) and trial type (familiar vs. novel pairing) was barely significant *F*(1, 18)  = 4.29, *p* = .052. This interaction reflected the fact that infants tended to look longer to the first test trial compared to the second one, although LSD post hoc tests did not show any significant difference. Infants therefore were not able to learn a rule that established a relationship between brightness/contrast level and number where larger numbers are accompanied by darker (or lower contrast) objects.

Further analyses compared infants’ looking patterns across Experiments 1 and 2. Infants showed similar looking times on the first three and last three familiarization trials across the two experiments, each *t*(38) <1, n.s., and they reached the habituation criterion after similar numbers of trials, *t*(38) <1, n.s. (unpaired-samples t-tests). Looking times for the pairing following the new rule at test did not differ significantly across the two experiments (*F*(1, 36)  = 2.65, *p* = .11; all other effects and interactions were not significant, all *F*s<3.25, *p*s >.08). Thus, although number-brightness pairings yield some positive results in Experiment 1 and not in Experiment 2, the two experiments together provide only weak evidence that infants are predisposed to map displays with greater numbers to forms with greater brightness or contrast levels.

Comparing the findings of Experiments 1 and 2 to those of de Hevia & Spelke (2010), there are two main differences that distinguish infants' responses to number-length and number-brightness pairings. First, the studies of number-length pairings revealed a significant difference between the performance of infants habituated to the positive pairings (i.e., larger numbers accompanied by longer lines) and those habituated to the inverse pairings (i.e., larger numbers accompanied by shorter lines). In the present studies using number-brightness pairings, in contrast, the difference between the two pairing directions was not significant. Second, although the two groups of infants habituated to one type of rule relating number-length and number-brightness pairings showed successful discrimination at test, with their looking times to the two test trials differing significantly, discrimination was manifested in opposite ways: Higher looking times were deployed for the familiar number-length test pairings, but for the novel number-brightness pairings. This qualitative difference suggests that infants at this age give a different treatment to the two types of pairings involving numerical information on the one hand and the dimensions of length and brightness on the other.

## Experiment 3

Nevertheless, it is possible that infants' sensitivity to number-brightness relations is greater than Experiments 1 and 2 suggest: this preference may be obscured by a strong baseline preference for pairings where larger numbers are associated with darker objects. To investigate this possibility and test further for sensitivity to number-brightness relations, Experiment 3 investigated infants' baseline preferences for arrays in which numbers and levels of brightness are paired in both directions: larger numbers paired with brighter or with darker forms. The experiment used the method of de Hevia & Spelke (2010, Exp. 4): a method that revealed at this age a preference for positive number-length pairings, in which larger numbers accompanied longer lines. We ask whether infants also show a preference for either type of number-brightness pairings when tested with this method.

### Method

A new group of 20 infants (16 female and 4 male; mean age  =  8 months, range: 7 months 17 days to 8 months 15 days) participated in this experiment. One additional infant was excluded for crying. The method was the same as in Experiments 1 and 2 except that no familiarization sequences were presented, and the two test displays appeared three times in alternation for a total of six test trials. Looking times were analyzed by an ANOVA with test-trial pair (first, second, or third pair) and test display (larger-brighter vs. larger-darker pairing) as within-subjects variables and test order (larger-brighter pairing first vs. second) as a between-subjects variable.

### Results and Discussion

Infants looked equally long to both pairings, *F*(1, 18)  = 1.48, *p* = .24, but their looking times differed significantly across the three pairs of trials, *F*(2,36)  = 4.49, *p* = .01, and this effect was complicated by a significant three-way Test-Trial Pair × Test Display × Test Order interaction, *F*(2, 36)  = 6.03, *p*<.01. LSD post hoc tests revealed a preference for the first trial pair, with a preference for the first positive pairing among infants who received the positive pairing first (*p* = .01), and a preference for the first inverse pairing among infants who received that pairing first (*p* = .03; all other *p*s >.13). Therefore, infants looked longer at the first test trial irrespective of the pairing that it displayed.

**Figure 3:**
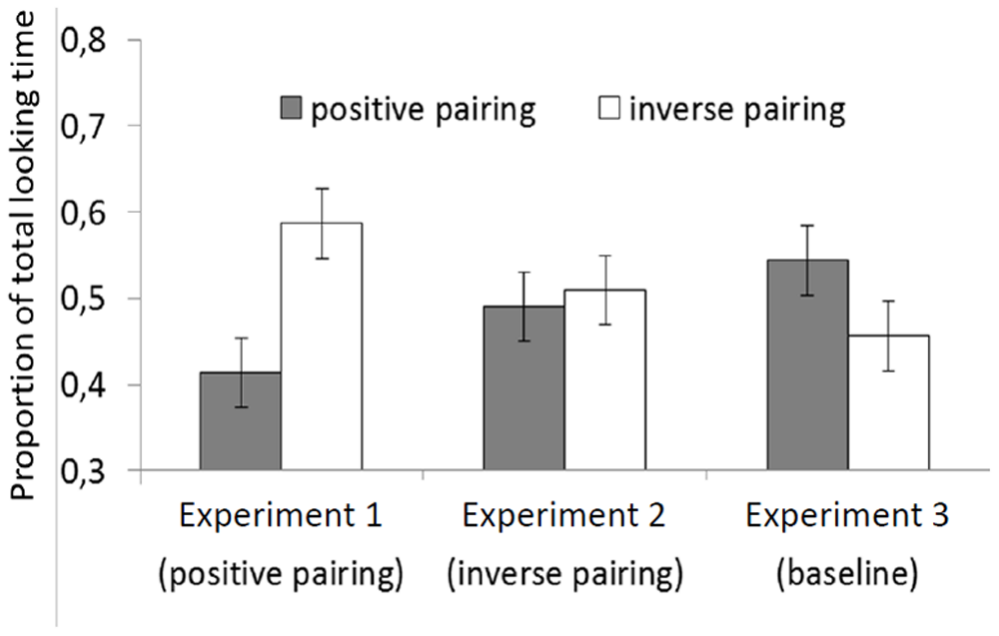
Percentage of looking time towards positive and inverse number-brightness pairings across Experiments 1, 2 and 3. Proportion of looking time towards the positive and inverse number-brightness pairings across experiments: Experiment 1 (positive pairing), Experiment 2 (inverse pairing), and Experiment 3 (baseline condition with no familiarization).

In order to compare performance from Experiments 1 and 2 against baseline performance, we transformed the raw test data from all three experiments into percentages of total looking times, and entered these data into two separate one-way ANOVAs, testing for differences in looking times to positive pairings and to inverse pairings for each Experiment compared to baseline. The analyses showed a significant difference between Experiment 1 and the baseline condition (both *F*s(1,38)  = 5.27, *p*s = .02), with higher looking to the positive pairing in the baseline condition than in Experiment 1. The analyses did not reach statistical significance for the comparisons between the baseline condition and Experiment 2 (both *F*s<1, n.s.; Fig. 3). These analyses provide some evidence that infants learned the number-brightness rule where larger numbers were accompanied by brighter objects (Experiment 1), and no evidence that they learned the number-brightness rule where larger numbers were accompanied by darker objects (Experiment 2). Because learning of the positive and inverse rules did not differ significantly, however, these findings provide only partial evidence that infants are predisposed to relate changes in number to changes in brightness.

## Experiment 4

Since infants showed some evidence of learning a number-brightness rule where larger numbers are accompanied by brighter objects, Experiment 4 tested whether infants transfer the discrimination of an ordered series of numbers to an ordered series of brightness and contrast levels. As in de Hevia & Spelke (2010), 8-month-old infants were habituated to one series of five visual dot arrays presenting either successive increases or successive decreases in number. Then all infants were presented with six alternating trials of increasing and decreasing levels of brightness, and their looking times towards the displays were measured. If infants treat all continuous dimensions equally, then they should generalize the ordinal direction from the number to the brightness displays, as they do for the dimensions of number and length [32].

### Materials and Methods

#### Participants

A new group of 24 full-term infants (14 female and 10 male; mean age  =  8 months, range: 7 months 16 days to 8 months 13 days) participated in the experiment. Four other infants were eliminated because of crying (2), or test-trial looking times more than 3 standard deviations from the overall group mean (2; results did not change when these two participants were included).

#### Materials

Numerical displays were identical to those employed in the previous experiments, but at double size, and they were presented centered against a black background and occupying the entire screen. Summed area was equated across displays by varying item size inversely to number: For arrays of 4, 8, 16, 32, and 64, respectively, circle diameters were 3.8°, 2.7°, 1.9°, 1.3°, and 0.9° visual angle; square sides were 3.4°, 2.4°, 1.7°, 1.2°, and 0.8° visual angle; and triangle sides were 4.8°, 3.4°, 2.4°, 1.7°, and 1.2° visual angle. We held envelope area constant across displays by positioning items randomly within a fixed area. Test displays consisted of the same cross as in the previous experiments, centrally positioned on the screen.

**Figure 4:**
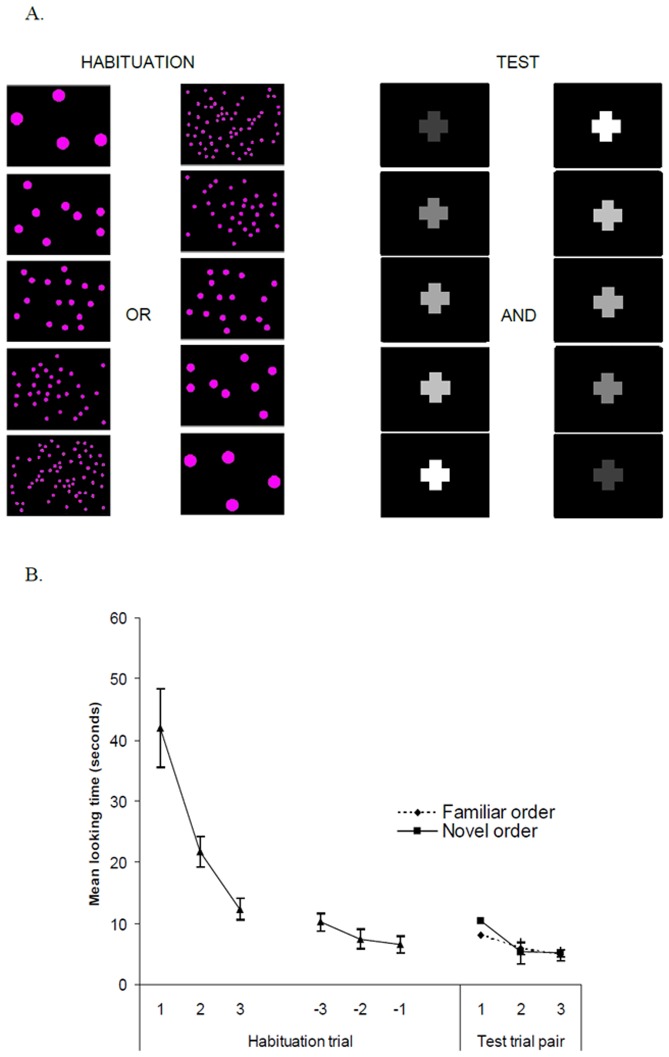
Stimuli used in the familiarization and test phases for Experiment 4, and mean looking times habituation and test trials. A. Example of stimuli in the habituation phase where infants are habituated to either increasing or decreasing number, and test trials where all infants are tested with both increasing and decreasing brightness levels. B. Mean looking times (seconds) towards the first three, the last three habituation trials, and to the familiar and novel order test trials across pairs of trials. No difference was observed in the looking times between familiar and novel test trials.

Each numerical trial consisted of a repeating cycle (9 s in total) that began with the image of a dog moving while noise was played (1000 ms). After a blank screen (500 ms), a series of five numerical displays (1200 ms each) was presented. Each numerical display was followed by a blank screen (300 ms; total sequence length  =  7500 ms). Brightness trials were identical to the numerical trials, except that the displays consisted of the cross at different brightness levels (Fig. 4A).

#### Design

As in de Hevia & Spelke (2010), and prior to the habituation trials, infants were familiarized with the displays to appear in the test trials. During this phase, all infants were presented with two familiarization trials showing the same increasing and decreasing brightness levels that would be presented later at test. As in de Hevia & Spelke's experiments (2010), these trials were included so that looking patterns during the test would not be overwhelmed by a general novelty reaction to the presentation of a new type of display. Half of the infants were then randomly assigned to each of the two habituation conditions: They were habituated to either an ascending numerical sequence or a descending numerical sequence. The order of the two familiarization trials (increasing vs. decreasing sequences) and of the test trials (familiar first vs. novel first) was counterbalanced across infants within each habituation group.

#### Procedure

First, infants were presented with two familiarization trials, which consisted on each of the two brightness-levels (test) displays. Each of these familiarization trials was visible until the infant had looked for 20 s. For the habituation and the test trials, the procedure was the same as in the previous experiments. Following habituation, all infants were shown 6 trials in which the two test displays appeared in alternation. For 19 out 24 infants, two observers coded the data live or from videotape; average intercoder reliability was 92%.

#### Analyses

Infants’ looking times during test trials were submitted to an analysis of variance (ANOVA) with habituation condition (ascending vs. descending) and test order (familiar first vs. second) as between-subjects variables, and test-trial type (familiar vs. novel) and test pair (one vs. second vs. third) as within-subjects variables. All other tests were two-tailed.

### Results and discussion

Infants spent a comparable time looking at the test trials with the congruent test displays presenting the same order of brightness levels (6.3 s), relative to the test trials with the incongruent test displays presenting the reversed order of brightness levels (7 s), *F*(1, 20) <1, n.s. (Fig. 4B). The only significant main effect was test pair, *F*(2, 40)  = 9.36, *p*<.001: infants showed higher looking times during the first pair of trials compared to both the second and third pairs (each *p*<.01, LSD post-hoc tests). No interactions were significant. Although infants successfully habituated to the ordered numerical sequences, with looking times during the last three habituation trials significantly shorter (24.3 s) than to the first three habituation trials (76.2 s; *t*(23)  = 7.94, *p*<.0001, paired-samples t-test; with an average of 7.5 habituation trials per participant), their looking times in the test trials showing the reversed ordering of brightness levels (21.1 s) did not significantly differ from their looking time to the last three habituation trials (*t*(23)  = 1.18, *p* = .3, paired-samples t-test). Infants therefore failed to generalize habituation from an increment or decrement in number to an increment or decrement in brightness (or contrast) level.

Although infants revealed some sensitivity to number-brightness relations when both dimensions appeared concurrently (Experiments 1 and 3), they failed to transfer discrimination from one dimension (number) to the other (brightness/contrast).

## General Discussion

This study investigated whether 8-month-old infants, who have been shown to successfully detect and learn number-length mappings, show the same ability for mappings between number and a different continuous dimension, level of brightness. In Experiment 1, infants presented with pairings between numbers and levels of brightness, with larger numbers associated to brighter objects, were able to establish a number-brightness mapping, and they productively used this mapping at test to differentiate it from a mapping not conforming to the positive rule. Moreover, their performance in Experiment 1 contrasted significantly from their performance in Experiment 3 (baseline), where infants did not have the opportunity to learn any mapping rule. In contrast, in Experiment 2, where number-brightness pairings were inversely related, such that larger numbers were associated to darker objects, infants failed at learning them, and their performance did not differ from the baseline condition of Experiment 3.

Nevertheless, infants express their ability to create number-brightness pairings differently from number-length pairings, tested with the same methods in previous research [32]. First, whereas infants given number-length pairings, where larger numbers were paired with longer lines, looked preferentially at familiar pairings at test, for number-brightness pairings infants looked preferentially at novel test pairings. Second, performance from infants in number-length mappings differed significantly depending on the rule relating the two dimensions (i.e., positive or inverse), whereas for number-brightness mappings there was no significant difference in performance for the two rules. This finding suggests that while number-length mappings are exclusively formed in a specific direction, this signature does not apply clearly to number-brightness mappings. Finally, when shown both larger-longer and larger-shorter pairings without previous familiarization, infants showed a baseline preference for the larger-longer number-length pairings. In the present study, on the contrary, infants looked longer at the first trial pair irrespective of which number-brightness pairing they were presented with (Experiment 3).

Finally, Experiment 4 tested infants’ ability to transfer discrimination from the dimension of number to the dimension of brightness. Infants failed at this task, suggesting that the representation of an ordered series of numerical quantities does not spontaneously link to the representation of an ordered series of brightness levels. In previous research, using the same methods and testing infants at the same age, a successful transfer from number to length was reported [32]. This finding points to possible differences in processing and/or representation of the two dimensions, number and brightness, providing evidence against the view that link any continuous dimension to any other in similar ways and with equal ease.

The pattern of findings reported in this study therefore suggests that number-length mappings and number-brightness mappings are treated differently by infants, supporting the view that some dimensions might share more privileged links than others by 8 months of age [6], [36]. One possible explanation for this finding is that the association between number and spatial extent derives from and/or becomes reinforced by exposure to the natural co-occurrences in the environment that emphasize their relationship, whereas number-brightness mappings might be naturally less common or salient. Also, if associations between magnitudes are made through action systems [23], [24], brightness might not be a relevant magnitude dimension to take into account during action-related computations at 8 months of age. Moreover, the present findings, together with previous evidence for successful mappings between brightness and loudness [35] and failure to map loudness and spatial length [34] might indicate that dimensions referring to the intensity of stimulation, such as brightness and loudness, are treated more similarly than the dimensions of number, spatial extent and time. Finally, another possibility is that mappings between quantitative dimensions are not learned during infancy through acting on the environment, but derive from biologically predisposed links between the dimensions of number, spatial extent and time: links that are functional early in infancy and possibly from birth. This idea, however, does not imply that infants are synesthetic from birth. Indeed, the evidence suggests that representations evoked by each of the dimensions of number, spatial extent and time are clearly differentiated from one another [39], despite the links between them.

In adults, the degree of anatomical overlap between processing of different dimensions correlates with differences in the degree of representational overlap between these dimensions. When adults see numbers and make judgments on orientation vs. color, stimulus attributes that respectively elicit more (orientation) or less (color) activity in parietal cortex, only orientation judgments are influenced by the numbers, when the numbers are irrelevant to the task at hand [7], [8]. Similarly, the anatomical proximity between the neural structures activated by the relevant and irrelevant dimensions in a Stroop paradigm can predict the amount of behavioural interference experienced by adults. For example, brightness and size anatomically overlap at occipito-temporal and posterior parietal regions, but only number and size also overlap at the level of the posterior intraparietal sulcus, and there is no posterior region overlap between number and brightness [6]. Consistent with these anatomical findings, behavioral experiments reveal that number and size, and size and brightness both show bidirectional interference, whereas no interference is found for brightness and number [6]. Thus, behavioral and anatomical evidence in adults shows that the coding of number and spatial extent share common neural resources at the level of the intraparietal sulcus, with these dimensions converging at an abstract representational level that is not shared by other perceptual dimensions such as brightness [6], which involve the visual ventral stream [40].

In our study, infants who observed monotonic increases and decreases of both number and brightness, showed some ability to learn a positive pairing between both dimensions when they appeared concurrently (in Experiment 1), but failed to transfer discrimination from one dimension to the other when they appeared successively (in Experiment 4). In contrast, when spatial length replaces brightness in this task, infants at the same age succeed at both tasks [32]. This pattern of findings supports the view that some quantitative dimensions share stronger links, in the sense of functional overlap, than other dimensions. Future research on the early anatomical basis for comparing quantitative dimensions might shed light on the observed degree of functional overlap across dimensions in childhood and infancy.

This study adds to previous evidence of stronger mappings between length and time than between length and loudness [34], by showing that infants’ mappings between number and length operate differently from mappings between number and brightness or contrast. Future research might fruitfully investigate whether brightness-length mappings are created similarly to number-length mappings, as some adults’ behavioral and anatomical evidence suggests [6]. Infants’ understanding of quantitative dimensions and of their relations can offer rich insight into how the cognitive and anatomical systems subserving magnitude processing develop and are organized.
